# Supplementary material for: Pyruvate Dehydrogenase Kinase 1 inhibition mediated oxidative phosphorylation enhancement in cartilage promotes osteoarthritis progression
Source: BMC Musculoskelet Disord. 2023 Jul 20;24:597. doi: 10.1186/s12891-023-06585-6 (PMC10357736; doi:10.1186/s12891-023-06585-6)
Supplement: Supplementary file 1 — Additional file 1. [file 12891_2023_6585_MOESM1_ESM.zip › BP.Gsea.1653623667859/GOBP_RIBOSOMAL_LARGE_SUBUNIT_ASSEMBLY.html]

Details for gene set GOBP\_RIBOSOMAL\_LARGE\_SUBUNIT\_ASSEMBLY[GSEA]

|  || Dataset | 55457\_collapsed\_to\_symbols.cls.cls #OA\_versus\_normal.cls.cls #OA\_versus\_normal\_repos |
| Phenotype | cls.cls#OA\_versus\_normal\_repos |
| Upregulated in class | normal |
| GeneSet | GOBP\_RIBOSOMAL\_LARGE\_SUBUNIT\_ASSEMBLY |
| Enrichment Score (ES) | -0.7115555 |
| Normalized Enrichment Score (NES) | -2.247434 |
| Nominal p-value | 0.0 |
| FDR q-value | 8.0954755E-4 |
| FWER p-Value | 0.002 |
Table: GSEA Results Summary

  

Fig 1: Enrichment plot: GOBP\_RIBOSOMAL\_LARGE\_SUBUNIT\_ASSEMBLY      
 Profile of the Running ES Score & Positions of GeneSet Members on the Rank Ordered List

  

| SYMBOL | TITLE | RANK IN GENE LIST | RANK METRIC SCORE | RUNNING ES | CORE ENRICHMENT || 1 | DDX28 | DEAD-box helicase 28 [Source:HGNC Symbol;Acc:HGNC:17330] | 4602 | 0.105 | -0.3461 | No |
| 2 | MRPL20 | mitochondrial ribosomal protein L20 [Source:HGNC Symbol;Acc:HGNC:14478] | 5989 | -0.004 | -0.4534 | No |
| 3 | NOP53 | NOP53 ribosome biogenesis factor [Source:HGNC Symbol;Acc:HGNC:4333] | 6334 | -0.031 | -0.4768 | No |
| 4 | RPL10L | ribosomal protein L10 like [Source:HGNC Symbol;Acc:HGNC:17976] | 6502 | -0.042 | -0.4852 | No |
| 5 | NLE1 | notchless homolog 1 [Source:HGNC Symbol;Acc:HGNC:19889] | 7253 | -0.099 | -0.5327 | No |
| 6 | FASTKD2 | FAST kinase domains 2 [Source:HGNC Symbol;Acc:HGNC:29160] | 7730 | -0.136 | -0.5550 | No |
| 7 | RPL3L | ribosomal protein L3 like [Source:HGNC Symbol;Acc:HGNC:10351] | 8097 | -0.163 | -0.5659 | No |
| 8 | DHX30 | DExH-box helicase 30 [Source:HGNC Symbol;Acc:HGNC:16716] | 8111 | -0.164 | -0.5493 | No |
| 9 | RPL5 | ribosomal protein L5 [Source:HGNC Symbol;Acc:HGNC:10360] | 10201 | -0.341 | -0.6749 | Yes |
| 10 | RPL24 | ribosomal protein L24 [Source:HGNC Symbol;Acc:HGNC:10325] | 10263 | -0.347 | -0.6422 | Yes |
| 11 | BRIX1 | biogenesis of ribosomes BRX1 [Source:HGNC Symbol;Acc:HGNC:24170] | 10275 | -0.348 | -0.6056 | Yes |
| 12 | MRTO4 | "MRT4 homolog, ribosome maturation factor [Source:HGNC Symbol;Acc:HGNC:18477]" | 10300 | -0.350 | -0.5698 | Yes |
| 13 | RPL10 | ribosomal protein L10 [Source:HGNC Symbol;Acc:HGNC:10298] | 10684 | -0.391 | -0.5574 | Yes |
| 14 | RRS1 | ribosome biogenesis regulator 1 homolog [Source:HGNC Symbol;Acc:HGNC:17083] | 11045 | -0.437 | -0.5384 | Yes |
| 15 | RPL38 | ribosomal protein L38 [Source:HGNC Symbol;Acc:HGNC:10349] | 11122 | -0.446 | -0.4963 | Yes |
| 16 | RPL6 | ribosomal protein L6 [Source:HGNC Symbol;Acc:HGNC:10362] | 11129 | -0.447 | -0.4487 | Yes |
| 17 | RPL11 | ribosomal protein L11 [Source:HGNC Symbol;Acc:HGNC:10301] | 11252 | -0.464 | -0.4082 | Yes |
| 18 | MDN1 | midasin AAA ATPase 1 [Source:HGNC Symbol;Acc:HGNC:18302] | 11973 | -0.588 | -0.4008 | Yes |
| 19 | PPAN | peter pan homolog [Source:HGNC Symbol;Acc:HGNC:9227] | 12022 | -0.597 | -0.3402 | Yes |
| 20 | RPLP0 | ribosomal protein lateral stalk subunit P0 [Source:HGNC Symbol;Acc:HGNC:10371] | 12285 | -0.672 | -0.2883 | Yes |
| 21 | RPL3 | ribosomal protein L3 [Source:HGNC Symbol;Acc:HGNC:10332] | 12350 | -0.691 | -0.2189 | Yes |
| 22 | NOP2 | NOP2 nucleolar protein [Source:HGNC Symbol;Acc:HGNC:7867] | 12372 | -0.699 | -0.1453 | Yes |
| 23 | BOP1 | BOP1 ribosomal biogenesis factor [Source:HGNC Symbol;Acc:HGNC:15519] | 12580 | -0.785 | -0.0769 | Yes |
| 24 | RPL23A | ribosomal protein L23a [Source:HGNC Symbol;Acc:HGNC:10317] | 12785 | -0.944 | 0.0089 | Yes |
Table: GSEA details [plain text format]

  

Fig 2: GOBP\_RIBOSOMAL\_LARGE\_SUBUNIT\_ASSEMBLY      
 Blue-Pink O' Gram in the Space of the Analyzed GeneSet

  

Fig 3: GOBP\_RIBOSOMAL\_LARGE\_SUBUNIT\_ASSEMBLY: Random ES distribution      
 Gene set null distribution of ES for **GOBP\_RIBOSOMAL\_LARGE\_SUBUNIT\_ASSEMBLY**
